# PTPN12 is a novel biomarker associated with genomic instability, therapeutic potentials, and immunomodulator in colorectal cancer

**DOI:** 10.3389/fphar.2026.1766515

**Published:** 2026-04-20

**Authors:** Siyi Qian, Han Gong, Peihe Zhang, Yiming Pan, Bin Zhang, Qiang Liu

**Affiliations:** 1 Department of Hepatobiliary and Intestinal Surgery of Hunan Cancer Hospital and the Affiliated Cancer Hospital of Xiangya School of Medicine, Central South University, Changsha, China; 2 Department of Histology and Embryology, Xiangya School of Basic Medical Sciences, Central South University, Changsha, China; 3 Department of Hematology, The Second Xiangya Hospital, Central South University, Changsha, China; 4 Department of anesthesiology, Third Xiangya hospital, Central South University, Changsha, China; 5 Institute of Blood Transfusion, Chinese Academy of Medical Sciences and Peking Union Medical College, Beijing, China

**Keywords:** biomarker, CRC (colorectal cancer), drug resistance, PTPN12/PTP-PEST, therapeutic target, tumor microenvironment

## Abstract

**Background:**

Protein tyrosine phosphatase non-receptor type 12 (PTPN12), a crucial enzymatic protein involved in cellular signaling, remains understudied in colorectal cancer (CRC). This study investigates this biological macromolecule’s potential as a biomarker, examining its protein-protein interactions, therapeutic relevance, and immunomodulatory functions in CRC.

**Methods:**

We conducted a comprehensive analysis of PTPN12 using public datasets and clinical samples. Bioinformatics tools were employed to predict regulators and signaling pathways associated with PTPN12. Immune infiltration analysis and re-analysis of publicly available single-cell RNA sequencing (scRNA-seq) datasets were performed to explore the immunomodulatory role of PTPN12. The relationship between PTPN12 protein levels and drug susceptibility was evaluated. Functional assays validated PTPN12’s role in CRC cells and its impact on oxaliplatin resistance.

**Results:**

PTPN12 protein is significantly upregulated in CRC tissues, with its elevated expression correlating with poor prognosis. PTPN12 correlates with increased genomic instability. PTPN12 is involved in various biological processes, including the regulation of cellular and developmental processes. Furthermore, high PTPN12 expression is positively correlated with stromal cell infiltration, suggesting a potential role in modulating the immune response. These findings collectively suggest that PTPN12 may have potential as a therapeutic candidate and immunotherapy-related biomarker. Knockdown of PTPN12 inhibited CRC cell proliferation, migration, and invasion. Notably, PTPN12 protein was overexpressed in oxaliplatin-resistant CRC cells, and its inhibition restored chemosensitivity in in vitro models.​

**Conclusion:**

PTPN12 shows promise as a potential biomarker and therapeutic target candidate in CRC. Our study provides preliminary insights into the role of PTPN12 in CRC pathogenesis, treatment response, and chemoresistance, which may lay the groundwork for future development of personalized therapeutic strategies pending further *in vivo* validation.

## 1 Introduction

Colorectal cancer (CRC) is currently the third most common and the second most deadly cancer globally (Morgan et al., 2023). The rising incidence and mortality rates of CRC, particularly in nations with lower socio-demographic indices, represent a significant public health challenge (Qu et al., 2022). Despite advancements in screening, diagnosis, and treatments, the prognosis for patients with advanced CRC remains poor (Song, 2022). Alarmingly, the rate of early-onset CRC in individuals under 50 has surged (Dekker et al., 2019; Patel et al., 2022). Therefore, identifying reliable biomarkers for CRC is crucial to enhance diagnostic and prognostic assessment.

Protein tyrosine phosphatases are critical modulators of tyrosine kinase–dependent signaling pathways and play essential roles in maintaining cellular homeostasis (Alonso and Pulido, 2016). Dysregulation of these phosphatases has been implicated in the development and progression of various malignancies (Sivaganesh et al., 2021; Zheng et al., 2008; Song et al., 2022). Protein tyrosine phosphatase non-receptor type 12 (PTPN12) is a member of the protein tyrosine phosphatase (PTP) family and has been shown to be a key regulator of EGFR/HER2 signaling (Li et al., 2016). Previous studies have reported that PTPN12 participates in tumor development and progression in multiple cancer types. For example, dysregulation of PTPN12 has been implicated in breast cancer, hepatocellular carcinoma, and nasopharyngeal carcinoma, where it influences tumor cell proliferation, migration, and signaling pathway activation (Cao et al., 2025; Neill et al., 2025; Lin et al., 2018; Liang et al., 2022). Mechanistically, PTPN12 is a non-receptor protein tyrosine phosphatase that negatively regulates oncogenic signaling by dephosphorylating multiple receptor and non-receptor tyrosine kinases. Previous studies have shown that PTPN12 can directly regulate substrates such as EGFR, HER2, Src family kinases, and focal adhesion kinase (FAK), thereby suppressing downstream pathways including RAS–MAPK and PI3K–AKT, which are critical for tumor cell proliferation and survival (Wang et al., 2023; Lee et al., 2020; Villa-Moruzzi, 2011). In addition, by modulating FAK–p130Cas–mediated focal adhesion signaling, PTPN12 regulates cytoskeletal dynamics and cell motility, processes closely associated with tumor invasion and metastasis (Hempel et al., 2013). In colorectal cancer, previous studies have mainly focused on the genetic susceptibility associated with PTPN12. Several genetic association analyses have identified PTPN12 as one of the genes associated with CRC risk (Belhadj et al., 2020; Brode et al., 2017; de Voer et al., 2016). While these findings suggest a potential involvement of PTPN12 in CRC development, its biological functions, molecular mechanisms, and clinical relevance in CRC remain largely unclear. Therefore, a comprehensive investigation integrating multi-omics analysis and functional validation may provide further insights into the role of PTPN12 in CRC progression and therapeutic response.

Beyond oncogenic signaling, PTPN12 has emerged as an important mediator of inflammation and immune regulation. In osteoarthritis, for example, the circ_0128846/miR-940/PTPN12 axis influences chondrocyte inflammation, implicating PTPN12 in inflammatory signal control (Fu et al., 2022). In immune cells, PTPN12 governs dendritic cell (DC) migration by regulating proline-rich tyrosine kinase 2 (Pyk2) phosphorylation—an essential step for DC trafficking from peripheral tissues to secondary lymphoid organs and for the initiation of T cell–dependent immune responses (Rhee et al., 2014). Collectively, these findings suggest that PTPN12 functions at the interface of oncogenic and inflammatory signaling, yet its specific roles in CRC remain poorly defined.

The tumor microenvironment (TME) is fundamental to cancer progression, encompassing interactions among cancer cells, stromal cells, and immune cells, significantly influencing CRC metastasis, immune escape, and resistance to therapy (Shin et al., 2023; Zhong et al., 2022; Ikeda et al., 2025). CRC develops within a chronically inflamed milieu: persistent colonic inflammation (e.g., in inflammatory bowel disease) and the infiltration of diverse immune populations—particularly DCs, macrophages, and T cells—render inflammatory signaling a defining hallmark of CRC pathogenesis (Schmitt and Greten, 2021). The nuclear factor kappa B (NF-κB) pathway, among others, serves as a critical bridge linking inflammation to tumor initiation and progression (Mao et al., 2025). Understanding how molecular regulators such as PTPN12 intersect with these inflammatory pathways may therefore uncover novel therapeutic and immunomodulatory targets.

Although PTPN12 has been implicated in the regulation of tumor growth and signaling in other cancers, its potential involvement in CRC’s distinct, inflammation-driven TME has not been investigated. This gap limits our understanding of how PTPN12 might modulate immune infiltration, inflammatory signaling (including NF-κB activity), or genomic instability within the CRC microenvironment.

This study aims to elucidate the role of PTPN12 in CRC, focusing on its association with genomic instability, therapeutic potentials, and, importantly, its immunomodulatory and inflammatory regulatory functions within CRC’s unique TME. Through the comprehensive analysis of public datasets and functional experiments, we aim to clarify how PTPN12 links inflammatory signaling to TME dynamics in CRC and explore its implications for CRC diagnosis and treatment.

## 2 Materials and methods

### 2.1 Data collection

Transcriptomic data from CRC patient samples, along with corresponding clinical information, were retrieved from repositories such as The Cancer Genome Atlas (TCGA) and Gene Expression Omnibus (GEO) databases. Only datasets with comprehensive clinical annotations and gene expression profiles were included in the analysis. The methylation level of PTPN12 in CRC was obtained through Illumina human methylation 450 array of the GDC portal (https://portal.gdc.cancer.gov/). The protein data of PTPN12 in pan-cancer was downloaded from the Clinical Proteomic Tumor Analysis Consortium (CPTAC) database (Li et al., 2023).

### 2.2 Genomic alteration analysis

The genomic alteration of PTP12 in pan-cancer (including Mutation, Structural Variant, Amplification, Deep Deletion, and Multiple Alterations) were analyzed by cBioPortal database (https://www.cbioportal.org/). The comparison of mutation and copy number variation between the high PTPN12 group and low PTPN12 group was performed by the BEST tool (https://rookieutopia.com/app_direct/BEST/).

### 2.3 Prognostic value of PTPN12

We assessed the prognostic relevance of PTPN12 in CRC using Kaplan-Meier (KM) method with optimal cutoffs (Gong et al., 2024; Duan et al., 2024). The Sangerbox 3.0 tool (Shen W. et al., 2022) was used to perform univariate Cox analysis for exploring the relationship between PTPN12 and overall survival (OS) across pan-cancer.

### 2.4 Upstream regulators analysis of PTPN12

The transcription factors of PTPN12 were predicted using four databases: hTFtarget (Zhang et al., 2020), Jaspar (Rauluseviciute et al., 2024), Cistrome (Zheng et al., 2019), and ENCODE (Consortium, 2012). A correlation analysis between the candidate TFs and PTPN12 expression was conducted employing the Spearman method. Differential methylation analysis between tumor and normal tissues was carried out using DiseaseMeth 3.0 (Xing et al., 2022).

### 2.5 Functional analysis of PTPN12 in CRC

The GeneMANIA tool (Franz et al., 2018) was utilized to construct the protein-protein interaction (PPI) network for PTPN12. To elucidate the potential role of PTPN12 in CRC, we initially identified the top 500 genes correlated with PTPN12 in CRC. Gene Ontology (GO) and gene set enrichment analysis (GSEA) methods were then conducted to investigate the biological functions associated with these genes.

### 2.6 Tumor microenvironment (TME) and scRNA analyses

The impact of PTPN12 expression on TME infiltration in CRC was evaluated using the ESTIMATE algorithm (Yoshihara et al., 2013), which provided immune and stromal scores. Additionally, the CAMOIP database (Lin et al., 2022), utilizing the MCPcounter algorithm, was employed to quantify the fractions of immune and stromal cells in the TCGA-CRC cohort. Furthermore, the TISCH2 database (Han et al., 2023) was utilized to annotate three single-cell RNA sequencing (scRNA-seq) datasets (EMTAB-8107, GSE146771, GSE166555) using established cell markers.

### 2.7 Potential drug screening

Utilizing the drug response and gene expression profiles of PRISM database (Co et al., 2020) for each compound, we employed the Spearman method to predict drug sensitivity across ten CRC datasets. Subsequently, we selected the top ten candidate agents with the most significant negative or positive correlations as potential therapeutic candidates.

### 2.8 Immunohistochemistry (IHC) analysis

We collected formalin-fixed paraffin-embedded (FFPE) samples from 98 CRC patients treated at Hunan Cancer Hospital (Changsha, China) between February 2016 and November 2020. The clinical characteristics of the patients are listed in Table 1. Pathological sections were dried in a desiccator at 60 °C–70 °C for 1 h, then rehydrated through an alcohol concentration gradient and placed in 0.01 M sodium citrate buffer at 100 °C for antigen retrieval. Endogenous peroxidase activity was blocked by incubation with 3% hydrogen peroxide solution at room temperature for 10 min. After three washes with PBS (3 minutes each), the slides were incubated with the primary antibody (1:100 dilution) at 4 °C overnight and then with the secondary antibody (Transgon, China) at room temperature for 20 min 3,3′-diaminobenzidine (DAB) staining and hematoxylin counterstaining were performed. The sections were examined under a microscope after dehydration through an alcohol gradient and coating with neutral resin.

**TABLE 1:** The clinical features of the patient.

Sample	Age	Gender	PTPN12 expression
1270689	<=65	Male	+
1264384	>65	Male	+
1265326	<=65	Male	-
1268620	<=65	Female	-
1252089	<=65	Female	+
1270688	<=65	Female	-
1273442	<=65	Male	-
1274385	<=65	Female	+
1274775	>65	Female	+
1276052	<=65	Male	+
1276053	>65	Male	+
1212538	<=65	Male	+
1207107	<=65	Male	+
1272650	<=65	Male	+
1256223	<=65	Male	-
1186973	<=65	Female	-
1145710	<=65	Male	-
1199772	>65	Female	+
1263255	>65	Male	+
1263943	>65	Male	+
1271995	>65	Female	-
1273574	<=65	Male	+
1274770	>65	Female	+
1125602	<=65	Female	+
1183449	<=65	Female	+
1255356	<=65	Male	-
1262637	>65	Male	+
1239206	<=65	Female	-
1265325	<=65	Male	+
1256807	<=65	Female	+
1262502	>65	Male	+
1276056	>65	Female	+
1201638	>65	Female	+
1254579	<=65	Female	+
1267932	<=65	Female	+
1126026	<=65	Male	-
1217406	<=65	Female	-
1267046	<=65	Male	+
1214826	<=65	Female	+
1240818	<=65	Male	-
1275463	>65	Male	+
1240496	<=65	Female	+
1271350	<=65	Female	+
1152270	>65	Female	+
1272364	<=65	Female	+
1273189	<=65	Male	+
1156961	<=65	Male	+
1179553	<=65	Male	+
1240349	>65	Male	+
1200841	<=65	Female	+
1152456	<=65	Male	-
1266962	>65	Male	+
1265347	>65	Male	-
1202184	>65	Female	+
1125317	<=65	Female	+
1114437	<=65	Male	-
1161405	<=65	Female	-
1267497	>65	Female	-
1267372	>65	Female	+
1268207	<=65	Female	+
1274409	>65	Female	+
1271099	<=65	Male	-
1259841	<=65	Male	-
1260270	<=65	Female	-
1260663	<=65	Female	+
1264487	<=65	Male	+
1267997	<=65	Male	+
1268318	>65	Female	+
1271594	<=65	Male	+
1273440	<=65	Female	+
1274949	>65	Female	+
1134179	<=65	Male	+
1194484	<=65	Female	-
1256525	<=65	Male	+
1202842	<=65	Male	-
1269272	<=65	Male	+
1117736	<=65	Female	+
1161144	<=65	Female	+
1213057	<=65	Female	+
1263258	<=65	Female	-
1170241	<=65	Male	-
1240908	<=65	Female	+
1159646	<=65	Male	+
1182658	>65	Male	+
1185079	<=65	Male	+
1268617	>65	Male	+
1270133	<=65	Male	+
1273338	<=65	Female	+
1273739	<=65	Female	+
1271530	<=65	Male	+
1254841	<=65	Male	+
1181845	>65	Female	+
1211761	<=65	Male	+
1211761	<=65	Male	-
1273448	>65	Male	-
1274461	<=65	Male	+
1135813	<=65	Female	+
1256984	<=65	Male	+

### 2.9 qRT-PCR and Western blot (WB)

qRT-PCR and WB experiments were performed according to our previous protocol. The primers were as follows:

GAPDH:

Forward: 5′-GTC​TCT​CTC​AAC​AGC​G-3′

Reverse: 5′-ACC​CCC​TGT​TGC​TGT​AGC​CAA-3′

PTPN12:

Forward: 5′-ACC​CGC​AGT​TGC​CTT​GTT​GAA​G-3′

Reverse: 5′-GTC​AAG​ATG​GGT​GGC​ACT​GGA​TG-3′

CD274:

Forward: 5′-CAT​TTG​CTG​AAC​GCC​CCA​TA-3′

Reverse: 5′- GGT​GAC​TGG​ATC​CAC​AAC​CA-3′

The primary antibodies as follows:

GAPDH: Servicebio, CN, GB15002-100

PTPN12: abcam, United Kingdom, ab76942

Bcl2: Wanleibio, CN, WL01556

Bax: Wanleibio, CN, WL01637

E-Cadherin: Wanleibio, CN, WL01482

N-Cadherin: Wanleibio, CN, WL01047

Vimentin: Proteintech, CN, 10366-1-AP

PD-L1: Wanleibio, CN, WL02778

### 2.10 Cell culture and transfection

The CRC cell lines HCT116, SW480, HCT116DR were purchased from the Cell Bank of the Chinese Academy of Science (Shanghai, China). These cells were cultured in DMEM medium (Gibco, United States), with all media enriched with 10% fetal bovine serum. streptomycin (Gibco, United States) at 37 °C in 5%CO2.

Plasmid Transfection: Taking one well of a six-well plate as an example, cells in the logarithmic growth phase were used for transfection when the cell density reached 60%–80%. Neofect transfection reagent was used for transfection, and the transfection reagent was prepared according to the manufacturer’s instructions: 100 μL pure DMEM culture medium, 2 μL Neofect transfection reagent, and 2 μg of the target plasmid were mixed, followed by incubation at room temperature for 15–30 min. After incubation, the mixture was added dropwise to the well containing 1.9 mL of complete culture medium. The cells were then transferred to a cell incubator and cultured for 36–48 h before performing transfection efficiency assays and subsequent experiments.

### 2.11 Cell proliferation assay

Cell viability was assessed using the Cell Counting Kit-8 (CCK-8, Biosharp, China) according to the manufacturer’s instructions. The number of viable cells was determined by measuring the absorbance at 450 nm.

### 2.12 Wound healing assay and transwell assay

Wound Healing Assay: Transfected cells were seeded in 6-well plates, and images were taken at 0, 24, and 48 h to monitor cell migration.

Transwell Assay: After 24 h of incubation, migrated cells were fixed and stained with 0.1% crystal violet. Invasion was quantified by counting the cells that invaded Matrigel and adhered to the lower membrane surface.

### 2.13 Cell apoptosis assay

For flow cytometric analysis of cell apoptosis, cell suspensions and cells digested with EDTA-free trypsin were collected and washed with pre-cooled phosphate-buffered saline (PBS). Cells were then stained using an apoptosis detection kit (Vazyme Biotech Co., Ltd., Nanjing, China) per the manufacturer’s instructions. After incubation at room temperature for 10 min in the dark, stained samples were analyzed via flow cytometry within 1 h to quantify the apoptotic rate.

### 2.14 Statistical analysis

Statistical analyses were performed using R software or GraphPad Prism 8. Statistical significance was assessed using the Student’s t-test or the Wilcoxon test, depending on the normality of the sample distribution. For comparisons involving multiple groups, the Kruskal−Wallis test was performed. Unless otherwise specified, a p-value of less than 0.05 was considered statistically significant.

## 3 Results

### 3.1 PTPN12 was highly expressed in CRC compared with normal tissues

**FIGURE 1:**
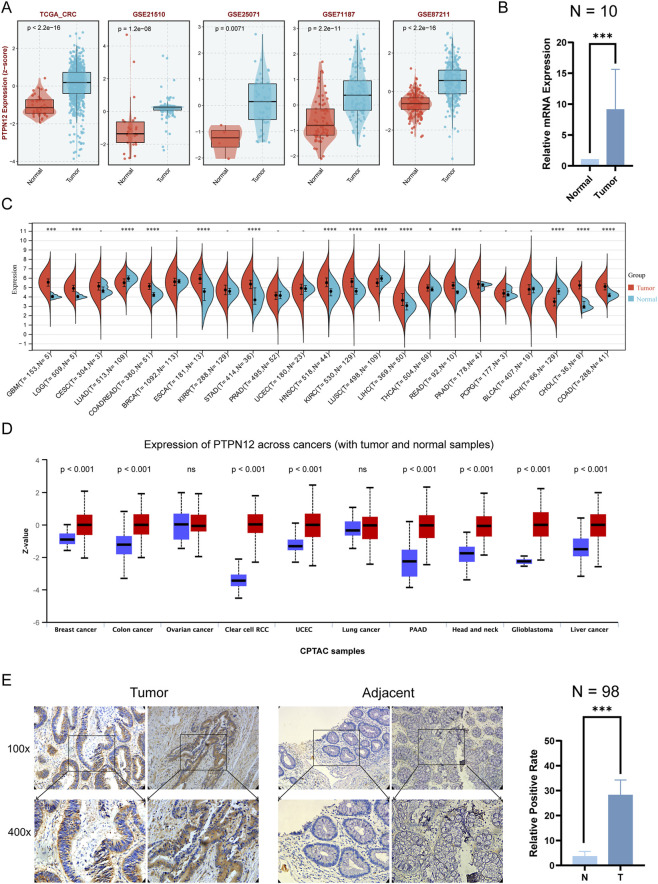
Differential expression of PTPN12 in normal and tumor tissues across multiple datasets. **(A)** Expression profiles from datasets TCGA-CRC, GSE21510, GSE25071, GSE71187, and GSE87211. Each panel shows a comparison between normal and tumor tissues within each dataset. **(B)** The mRNA expression of PTPN12 were elevated in clinical CRC samples. **(C)** A comprehensive analysis of PTPN12 expression across different cancer types from TCGA. This panel includes data from cancers such as GBM, LGG, CESC, and many others, comparing tumor (T) and normal (N) samples, with sample sizes indicated for each cancer type. **(D)** Differential analysis of PTPN12 protein levels across the Clinical Proteomic Tumor Analysis Consortium (CPTAC) database. Red represents tumor tissue, while blue represents normal control tissue. **(E)** Validation of PTPN12 protein expression in clinical samples analyzed by immunohistochemistry (IHC). *P < 0.05, ***P < 0.001, ****P < 0.001, and ns represents not significant.

We initially investigated the expression levels of PTPN12 in CRC. Differential analysis revealed that PTPN12 was significantly upregulated in five CRC datasets (TCGA-CRC, GSE21510, GSE25071, GSE71187, GSE87211) as illustrated in Figure 1A. We collected tumor samples and adjacent tissues from 10 CRC patients, and the qPCR results confirmed the significant upregulation of PTPN12 (Figure 1B). Moreover, pan-cancer analysis indicated that PTPN12 mRNA levels were elevated in 12 types of tumors, including Glioblastoma (GBM), Lower Grade Glioma (LGG), CRC, Esophageal Carcinoma (ESCA), Breast Invasive Carcinoma (BRCA), Stomach Adenocarcinoma (STAD), Head and Neck Squamous Cell Carcinoma (HNSC), Kidney Renal Clear Cell Carcinoma (KIRC), Liver Hepatocellular Carcinoma (LIHC), Thyroid Carcinoma (THCA), Rectum adenocarcinoma (READ), and Colon Adenocarcinoma (COAD), while showing downregulation in three tumors: LUAD, Lung Squamous Cell Carcinoma (LUSC), and Kidney Chromophobe (KICH) (Figure 1C). Protein expression analysis demonstrated that PTPN12 was upregulated in all examined tumors, except for lung and ovarian cancer (Figure 1D). To validate these findings using public datasets, 30 CRC clinical samples were collected and analyzed. Immunohistochemistry (IHC) analysis corroborated the high expression of PTPN12 (Figure 1E).

### 3.2 Genomic characteristics analysis of PTPN12

**FIGURE 2:**
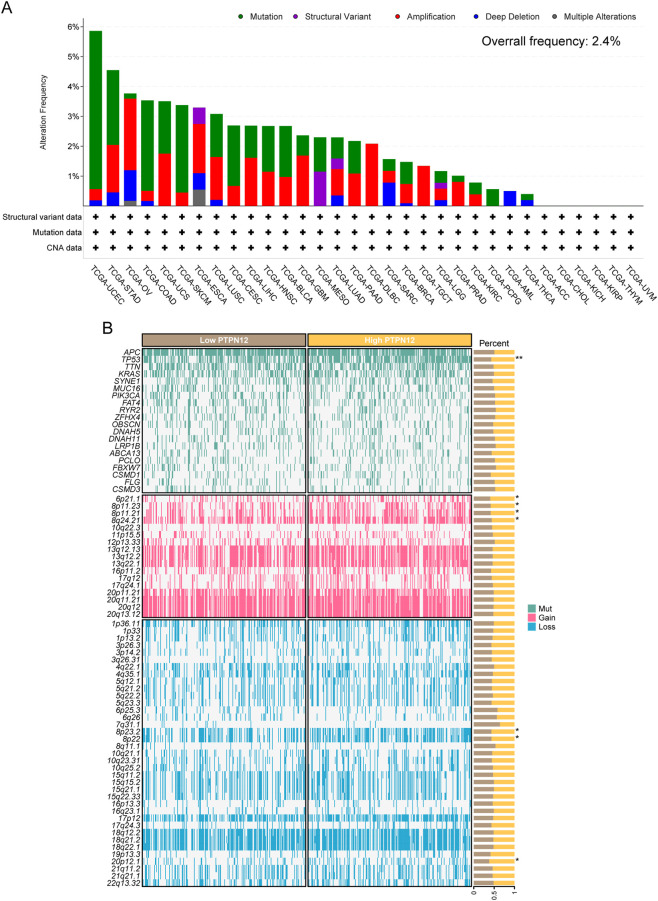
PTPN12 alteration profile across pan-cancer. **(A)** The alteration frequency of PTPN12 in pan-cancer from the cBioPortal database. **(B)** The genetic alterations with PTPN12 expression levels across different cancers, indicating whether higher or lower expression of PTPN12 is associated with mutations, gains, or losses in genes or loci. Green indicates gene mutation, red indicates copy number amplification, and blue indicates copy number deletion. The asterisks (*) indicate statistical significance of the findings. *P < 0.05 and **P < 0.01.

Cancer development and progression are intricately linked to genetic alterations (Yang et al., 2023; Berger and Mardis, 2018). In light of this, we explored the cBioPortal database for alterations in PTPN12, encompassing mutations, structural variants, amplifications, deep deletions, and multiple mutations (Figure 2A). The overall alteration frequency for PTPN12 was relatively low, at 2.4%, with uterine corpus endometrial carcinoma (UCEC) exhibiting the highest alteration frequency (∼5.8%). Additionally, we examined the genomic changes between patients with high and low PTPN12 expression. CRC patients with high PTPN12 expression exhibited a higher frequency of TP53 mutations, gains at chromosomal locations 6p21.1, 8p11.23, 8p11.21, and 8q24.21, and losses at 8p23.2, 8p22, and 20p12.1 (Figure 2B).

### 3.3 PTPN12 is a poor prognostic biomarker in CRC

**FIGURE 3:**
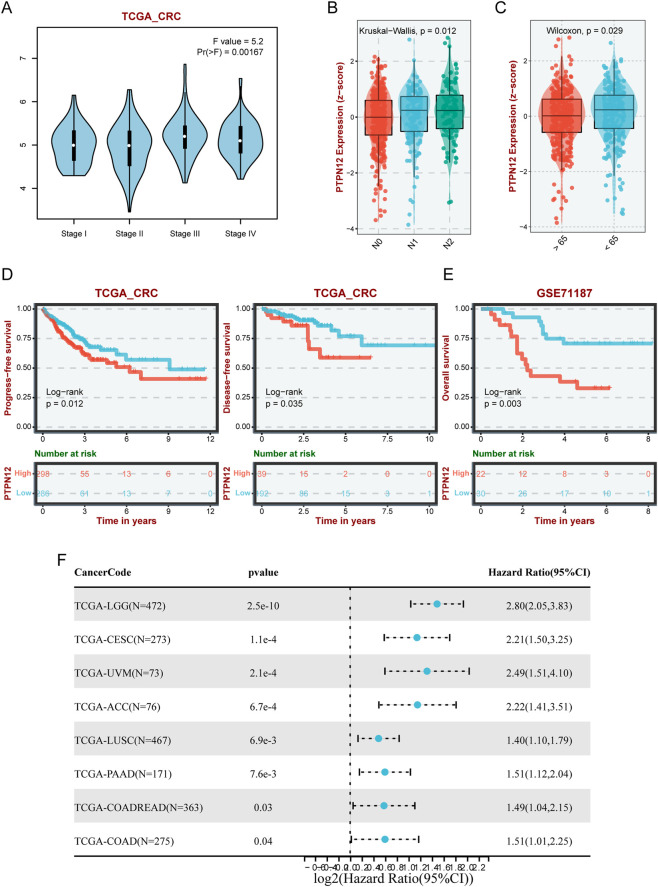
PTPN12 expression and its clinical implications in CRC. **(A)** PTPN12 expression across different cancer stages (I-IV) in TCGA-CRC dataset. **(B)** Expression levels of PTPN12 across nodal stages N0, N1, and N2 in CRC, showing a significant difference in expression (p = 0.012). **(C)** Comparison of PTPN12 expression in patients under and over 65 years old. **(D,E)** Kaplan-Meier plots illustrating overall survival in CRC patients with high vs. low PTPN12 expression from TCGA-CRC **(D)** and GSE71187 datasets **(E)**. **(F)** Forest plot summarizing the hazard ratios for progression-free survival in various cancer types from TCGA datasets, indicating increased risk associated with high PTPN12 expression.

To ascertain the clinical relevance of PTPN12 in CRC, we investigated its correlation with clinical characteristics. We found PTPN12 expression to be elevated in advanced stages in TCGA-CRC cohort (Figure 3A) and upregulated in N1 and N2 nodal stages (Figure 3B). Additionally, patients younger than 65 years old exhibited higher PTPN12 expression levels (Figure 3C). Survival analyses revealed an association between elevated PTPN12 levels and poorer progression-free survival (PFS) and disease-free survival (DFS) in TCGA-CRC cohort (Figure 3D). Consistent with our findings, an independent cohort (GSE71187) demonstrated that patients with high PTPN12 expression had poorer prognosis (Figure 3E). Univariate Cox regression analysis systematically examined the prognostic role of PTPN12 in pan-cancer. The forest plot showed that PTPN12 was a risk factor in eight types of tumors, including CRC (Figure 3F). Collectively, these findings suggest that PTPN12 is a potential biomarker for CRC and other malignancies.

### 3.4 Regulators analysis of PTPN12 upregulation in CRC

**FIGURE 4:**
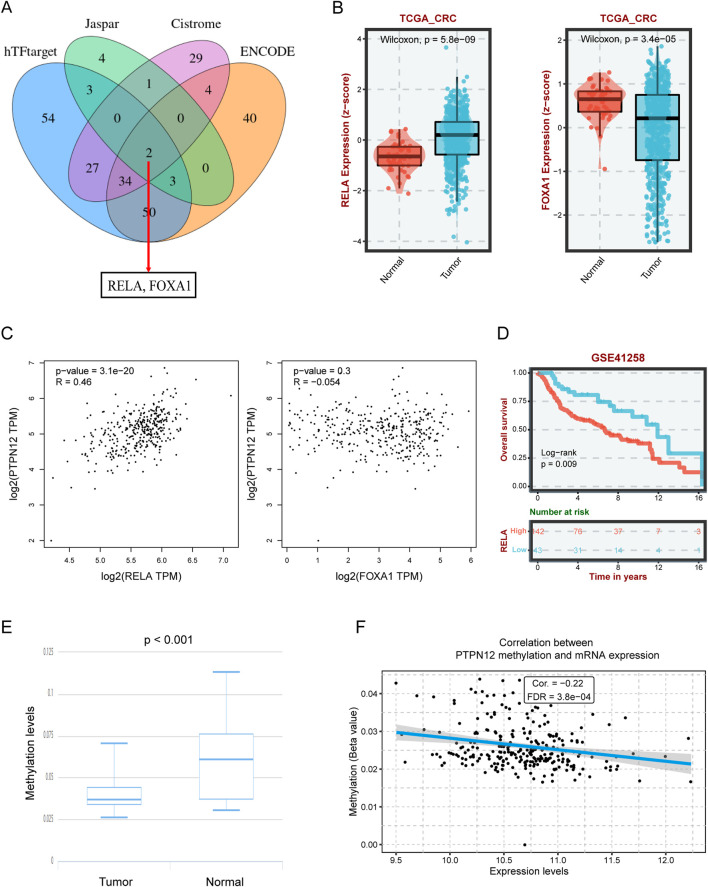
The regulation analysis of elevated PTPN12 in CRC. **(A)** The overlap of predicted transcription factors (TFs) from different databases. **(B)** Expression levels of RELA and FOXA1 in normal and tumor tissues from the TCGA-CRC dataset. **(C)** Scatter plot illustrating the correlation between RELA expression FOXA1 expression with PTPN12 expression. R: correlation coefficient. **(D)** Survival curves for CRC patients based on RELA expression levels (log-rank test, p = 0.009). **(E)** Caparison of PTPN12 methylation levels between tumor tissues and normal tissues in TCGA-CRC dataset. **(F)** Correlation analysis of methylation levels and PTPN12 mRNA expression. FDR: false discovery rate.

To investigate the upstream mechanisms responsible for PTPN12 dysregulation, we initially conducted a predictive analysis to identify potential transcription factors (TFs). By integrating data from four bioinformatics databases (hTFtarget, Cistrome, Jaspar, and ENCODE), we identified two candidate TFs: RELA and FOXA1 (Figure 4A). Differential expression analysis in the TCGA-CRC cohort revealed a significant upregulation of RELA and a notable downregulation of FOXA1 (Figure 4B). Correlation analysis further demonstrated a significant positive association between RELA and PTPN12 expression, whereas FOXA1 showed no significant correlation with PTPN12 (Figure 4C). Additionally, survival curves indicated that patients with elevated RELA levels had shorter survival times (Figure 4D), leading us to pinpoint RELA as a potential regulator of PTPN12.

In addition to transcriptional control, DNA methylation emerged as a crucial factor in gene dysregulation within tumors (Nishiyama et al., 2021). Comparative analyses of methylation levels between tumor and normal tissues revealed significantly lower methylation of PTPN12 in tumor samples (Figure 4E). Spearman’s correlation analysis further established a significant inverse relationship between methylation level and PTPN12 expression, underscoring the impact of epigenetic modifications on PTPN12 dysregulation (Figure 4F).

### 3.5 Potential mechanism analysis of PTPN12 in CRC

**FIGURE 5:**
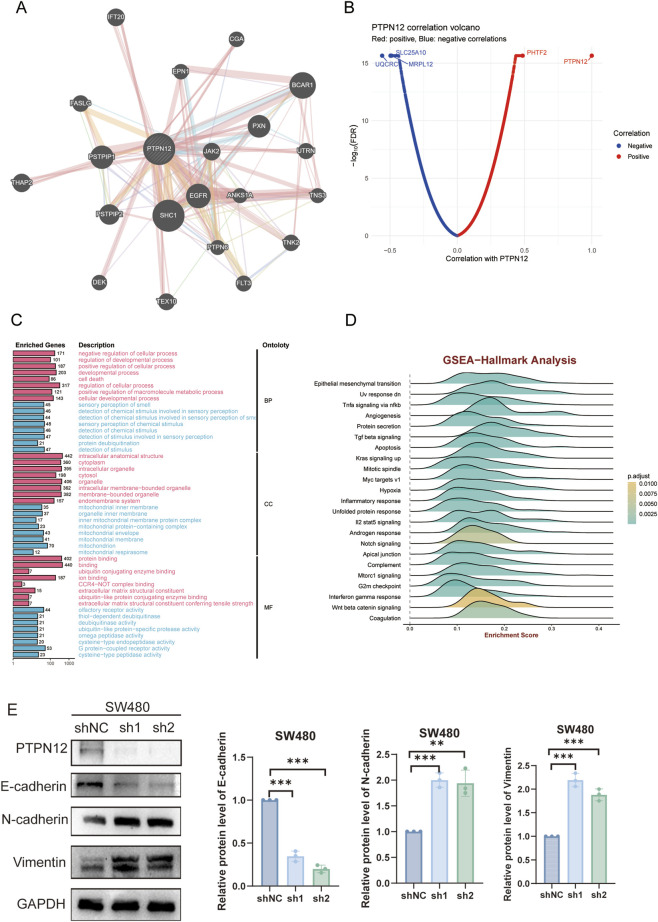
Functionally analysis of PTPN12 in CRC. **(A)** The protein-protein interaction (PPI) network of PTPN12 constructed by GeneMANIA. **(B)** Correlation and FDR-values of each gene with PTPN12 in the TCGA-CRC dataset. **(C)** Gene Ontology (GO) analysis of co-expressed genes related to PTPN12 expression, categorized into Biological Process (BP), Cellular Component (CC), and Molecular Function (MF). **(D)** Ridgeline plot showing the results of Gene Set Enrichment Analysis (GSEA). The positive scores indicate the extent to which hallmark pathways are enriched in CRC tissues with high PTPN12 expression. FDR: false discovery rate. **(E)** Western blot (WB) analysis of E-cadherin, N-cadherin, Vimentin in SW480 cells following PTPN12 knockdown.

Using the GeneMANIA database, we identified 20 proteins interacting with PTPN12. Analysis of the protein-protein interaction (PPI) network highlighted SHC1, PSTPIP1, PSTPIP2, BCAR1, and EGFR as hub genes (Figure 5A). Correlation analysis within the TCGA-CRC cohort revealed the top five genes most positively correlated with PTPN12 as PHTF2, LUZP6, SEPT7, OSBPL3, and OSTM1, and those most negatively correlated as UQCRC1, SLC25A10, MRPL12, CDX1, and ATP5D (Figure 5B).

We further performed GO enrichment analysis on the 500 most relevant genes to explore the potential role of PTPN12 in CRC (Figure 5C). Biological Process (BP) analysis indicated significant positive associations with developmental processes and negative associations with stimulus-response pathways. Cellular Component (CC) analysis suggested a positive correlation of PTPN12 with cytosolic components and a negative correlation with mitochondrial components. Molecular Function (MF) findings revealed a positive correlation with protein binding activities and a negative correlation with deubiquitinase activity. Furthermore, GSEA results demonstrated that cancer-related Hallmark gene sets were significantly enriched in high PTPN12 group, including pathways like epithelial-mesenchymal transition, TNFα signaling via NF-κB, and IL-2 STAT5 signaling (Figure 5D).

Among these, EMT is a well-characterized process driving CRC invasion, metastasis, and chemoresistance, prompting us to validate PTPN12’s role in regulating this pathway (Yang et al., 2025). We performed WB assays to detect the expression of key EMT markers (E-Cadherin, N-Cadherin, and Vimentin) in CRC cells after PTPN12 silencing. Results showed that PTPN12 knockdown led to a significant downregulation of E-Cadherin (an epithelial marker) and a marked upregulation of N-Cadherin and Vimentin (mesenchymal markers) (Figure 5E). These findings directly confirm that PTPN12 modulates the EMT pathway in CRC, providing a potential mechanistic link between PTPN12 overexpression and enhanced CRC aggressiveness.

### 3.6 The contribution of PTPN12 to tumor microenvironment in CRC

**FIGURE 6:**
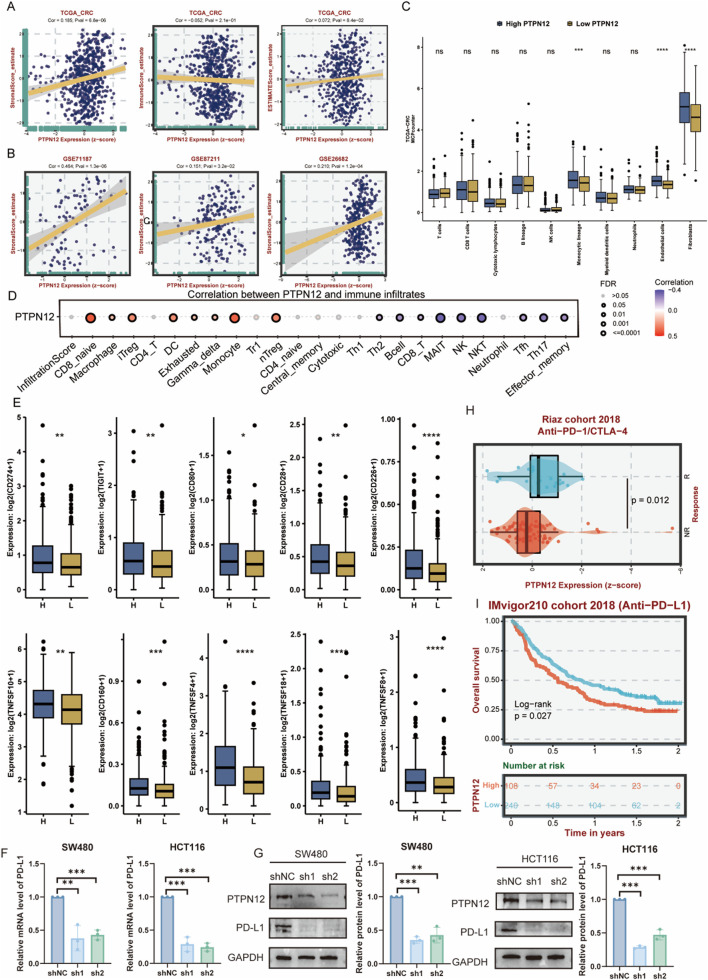
Impact of PTPN12 on tumor microenvironment in CRC and immunotherapy. **(A)** Correlation between PTPN12 expression with immune scores and stromal scores in the TCGA-CRC dataset, showing a positive correlation (Spearman method). **(B)** Additional correlations of PTPN12 expression with stromal scores in three independent datasets, further confirming the positive association with stromal components. **(C)** Comparison of the proportions of various immune and stromal cells between high PTPN12 expression and low PTPN12 expression groups in TCGA-CRC dataset. **(D)** Correlation between PTPN12 expression and immune cell infiltration. **(E)** Expression levels of immune checkpoint molecules (e.g., CD274, TIGIT, CD80, CD28, CD226, TNFSF8, TNFSF10, CD160, TNFSF4, TNFSF18) in relation to PTPN12 expression. **(F)** The qPCR analysis of PD-L1 in SW480 cells and HCT116 following PTPN12 knockdown. **(G)** Western blot (WB) analysis of PD-L1 in SW480 cells and HCT116 following PTPN12 knockdown. **(H)** Patients who response to immunotherapy exhibiting lower PTPN12 expression (p = 0.012). **(I)** Impact of PTPN12 expression on the efficacy of immune checkpoint blockade therapy (p = 0.027). *P < 0.05, **P < 0.01, ***P < 0.001, ****P < 0.001, and ns represents not significant.

The tumor microenvironment (TME) plays a crucial role in the pathogenesis, progression, and treatment response of CRC (Nenkov et al., 2021). Therefore, it is essential to comprehensively evaluate the impact of PTPN12 on the TME. Using the ESTIMATE algorithm, we evaluated the immune and stromal scores of CRC patients and found that PTPN12 expression was significantly positively correlated with stromal scores but not with immune scores (Figure 6A). This positive correlation between PTPN12 and stromal scores was further validated in three independent datasets (Figure 6B). To elucidate the impact of PTPN12 on specific cell types, we compared the proportions of infiltrating cells between PTPN12-high and PTPN12-low patient groups in the TCGA-CRC cohort. Notably, patients with high PTPN12 expression exhibited increased infiltration of monocytic lineage cells, endothelial cells, and fibroblasts (Figure 6C). The correlation heatmap further revealed that PTPN12 expression was positively correlated with regulatory T cells (Tregs), exhausted T cells, and naive CD8^+^ T cells, but negatively correlated with CD8^+^ T cells and natural killer (NK) cells (Figure 6D). Additionally, we assessed the expression patterns of immune checkpoints (ICs) between the two groups. The PTPN12-high group displayed significantly higher expression of immune checkpoint molecules (Figure 6E). Based on this observation, we further investigated whether PTPN12 could influence the expression of the key immune checkpoint ligand PD-L1 (CD274). Functional experiments were performed in CRC cell lines to validate this possibility. qRT-PCR analysis showed that knockdown of PTPN12 significantly reduced PD-L1 mRNA levels in both SW480 and HCT116 cells. Consistently, Western blot analysis confirmed that PD-L1 protein expression was markedly decreased following PTPN12 silencing (Figures 6F,G). These findings suggest that PTPN12 may regulate PD-L1 expression in CRC cells and thereby potentially participate in immune checkpoint modulation. In line with this potential role in immune regulation, we next evaluated the association between PTPN12 expression and immunotherapy outcomes. Intriguingly, patients who responded favorably to immunotherapy exhibited lower PTPN12 expression (Figure 6H). Survival analyses showed that patients with elevated PTPN12 expression had poorer outcomes following immunotherapy (Figure 6I). This underscores the necessity for targeted therapeutic strategies that consider the complex interplay between PTPN12 and the TME in CRC.

### 3.7 scRNA analysis of PTPN12 in CRC

**FIGURE 7:**
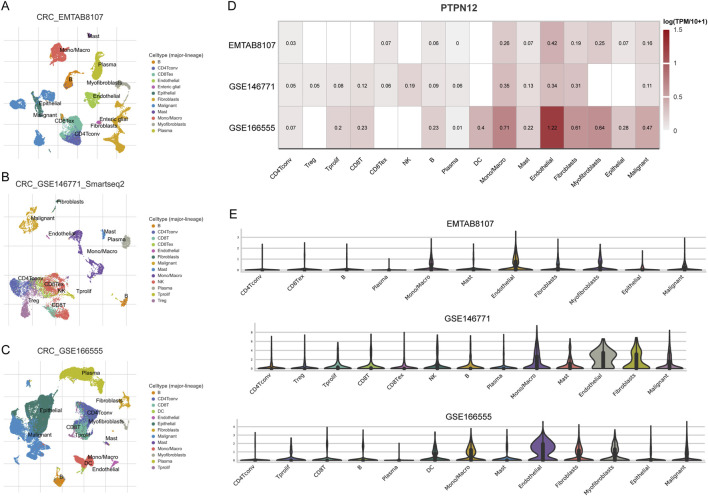
Expression analysis of PTPN12 across various cell types in CRC. **(A–C)** Identification of various cell types in CRC tissues. **(D)** Visualization of PTPN12 expression levels across different cell types within the tumor microenvironment of CRC, analyzed from multiple datasets including GSE166555, GSE146771, and EMTAB8107. **(E)** Violin plot of PTPN12 expression for each cell type in each dataset.

To delve deeper into the role of PTPN12 within the TME at the single-cell level, we utilized single-cell RNA sequencing (scRNA-seq) to examine its expression across three CRC cohorts (EMTAB8107, GSE146771, GSE166555). We annotated cells based on classical markers, identifying populations such as CD8 T cells, B cells, macrophages, fibroblasts, endothelial cells, and tumor cells (Figures 7A–C). Heatmap and violin plot analyses demonstrated relatively high PTPN12 expression in myofibroblasts, fibroblasts, and endothelial cells, whereas immune cell subsets such as natural killer (NK) cells and B cells exhibited lower PTPN12 levels (Figures 7D,E). These scRNA-seq data suggest a potential role for PTPN12 in the stromal and vascular components of the tumor microenvironment, corroborating our previous findings from bulk RNA-seq analyses.

### 3.8 Treatment guidance for CRC patients

**FIGURE 8:**
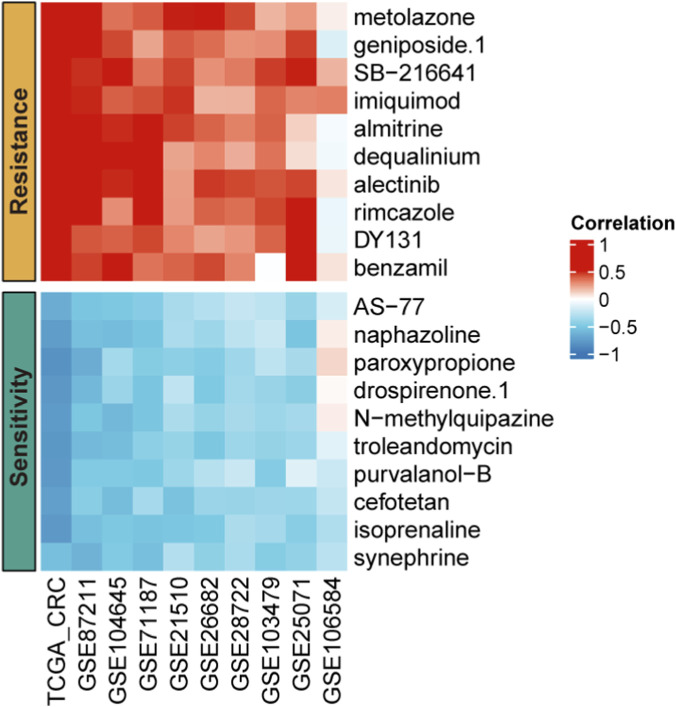
Drug sensitivity and resistance related to PTPN12 Expression. Overview of correlation of PTPN12 expression with drug sensitivity across multiple CRC datasets, including TCGA-CRC, GSE87211, GSE104645, GSE71187, GSE21510, GSE26682, GSE28722, GSE103479, GSE25071, GSE106584. Detailed correlation values ranging from −1 (indicative of sensitivity) to +1 (indicative of resistance).

Targeted therapy remains the mainstay of treatment for CRC, especially for patients with advanced stages (Piawah and Venook, 2019; Modest et al., 2019). To improve therapeutic strategies and personalize treatment, we performed drug response prediction based on PTPN12 expression levels across nine CRC cohorts. Utilizing pharmacogenomic data from the PRISM database, we calculated the sensitivity of CRC patients to various anti-tumor drugs. Correlation heatmap analysis revealed that patients with high PTPN12 expression exhibited resistance to metolazone, geniposide, SB-216641, imiquimod, almitrine, dequalinium, alectinib, rimcazole, DY131, benzamil, and AS-77, while displaying increased sensitivity to naphazoline, paroxypropione, drospirenone, N-methylquipazine, troleandomycin, purvalanol-B, cefotetan, isoprenaline, and synephrine (Figure 8). The consistent association between PTPN12 expression and drug response across multiple independent cohorts provides insights into potential therapeutic strategies based on PTPN12 status in CRC patients.

### 3.9 Functional experiments of PTPN12 in CRC cell lines

**FIGURE 9:**
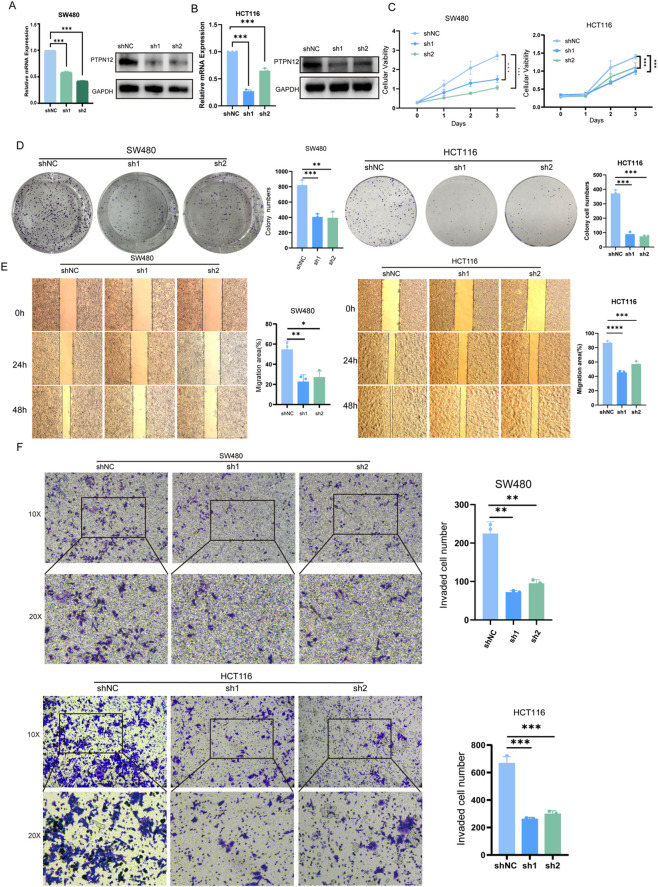
Effects of PTPN12 knockdown on the proliferation, migration and invasion functions of CRC cells. **(A,B)** The qPCR and Western blot (WB) analysis of PTPN12 in SW480 cells **(C)** and HCT116 cells **(B)**. **(C)** Cell proliferation assessed using the CCK-8 assay. **(D)** Colony formation assay showing the number of colonies in SW480 and HCT116 cells transfected with shRNA compared to the control. **(E)** Cell migration assessed by wound healing assay. **(F)** Cell invasion measured using the transwell invasion assay. *P < 0.05, **P < 0.01, ***P < 0.001, ****P < 0.001.

To explore the functional role of PTPN12 in colorectal cancer (CRC), we conducted knockdown experiments in two widely used and representative CRC cell lines, SW480 and HCT116. We performed the Quantitative PCR (qPCR) and Western blot (WB) assays to confirmed that the PTPN12 expression was significant downregulated compared to the control group both in mRNA and protein levels (Figures 9A,B). Subsequently, we assessed the impact of PTPN12 knockdown on cell proliferation. Both CCK-8 and colony formation assays indicated a notable reduction in cell proliferation and viability in the knockdown group compared to control cells (Figures 9C,D). Additionally, to evaluate the effect of PTPN12 on CRC cell migration and invasion, we performed wound healing and transwell assays. The results demonstrated a significant decrease in migration and invasion abilities following PTPN12 knockdown (Figures 9E,F). These findings provide valuable insights into the mechanisms through which PTPN12 promotes CRC progression.

### 3.10 PTPN12 inhibition attenuates the oxaliplatin resistance of CRC cells

**FIGURE 10:**
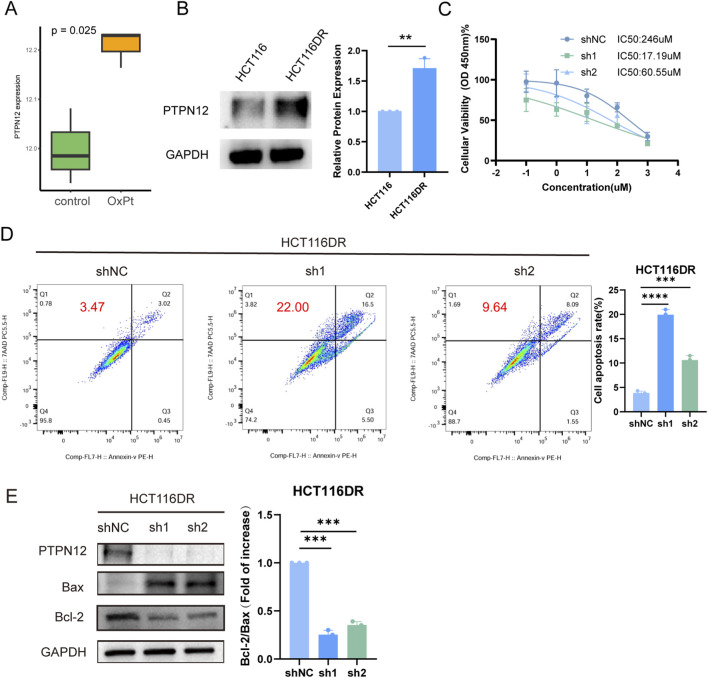
The impact of PTPN12 in oxaliplatin resistance of CRC cells. **(A)** Comparison of PTPN12 expression levels between HCT116-OxPt (oxaliplatin-resistant) and parental HCT116 cells. **(B)** Western blot analysis of PTPN12 protein levels in HCT116DR (oxaliplatin-resistant) cells. **(C)** Oxaliplatin sensitivity of HCT116DR cells following PTPN12 knockdown, as measured by cell viability assays. **(D)** Flow cytometry analysis quantifying apoptosis in HCT116DR cells after PTPN12 knockdown. **P < 0.01, ***P < 0.001, ****P < 0.0001. **(E)** Western blot (WB) analysis of Bcl2 and Bax in HCT116DR cells following PTPN12 knockdown.

Oxaliplatin, a platinum-based chemotherapeutic agent, is widely used as a first-line treatment for CRC. However, the development of drug resistance in some patients leads to treatment failure or disease recurrence (Shen X. et al., 2022; Hsu et al., 2018). Building upon our previous findings, we sought to investigate the potential association between PTPN12 and oxaliplatin resistance in CRC. We analyzed PTPN12 expression in drug-resistant cell lines using the publicly available dataset GSE42387. Our results revealed significantly higher PTPN12 expression in oxaliplatin-resistant cells (Figure 10A). To validate this finding, we conducted WB analysis on the drug-resistant cell line HCT116DR, which confirmed elevated PTPN12 expression (Figure 10B). Furthermore, cell viability assays using CCK-8 demonstrated that PTPN12 knockdown restored oxaliplatin sensitivity (Figure 10C). Flow cytometry analysis indicated that PTPN12 silencing effectively increased apoptosis in HCT116DR cells (Figure 10D). And WB analysis was performed to detect the apoptosis-related proteins Bcl-2 and Bax. The results showed that PTPN12 silencing led to a significant decrease in the Bcl-2/Bax ratio, which is indicative of increased cellular apoptosis (Figure 10E). In summary, our findings suggest that inhibition of PTPN12 appears to sensitize resistant cells to oxaliplatin treatment, potentially offering a new strategy to overcome chemoresistance in CRC.

## 4 Discussion

Colorectal cancer (CRC) poses a significant global health challenge and imposes a substantial burden on affected individuals and healthcare systems. While early-stage CRC can be effectively treated with surgical intervention, the 5-year survival rate for metastatic colorectal cancer remains alarmingly low, at less than 15%. Notably, approximately 20% of newly diagnosed CRC cases present with metastatic disease at the time of diagnosis (Biller and Schrag, 2021). Concerningly, the incidence of CRC among younger individuals is on the rise, further underscoring the urgent need to develop novel therapeutic targets and identify reliable biomarkers for enhanced risk stratification and screening (Lech et al., 2016).

PTPN12 is ubiquitously expressed across various tissues and plays a critical role in maintaining cellular homeostasis through the regulation of multiple signaling pathways. In this study, we systematically analyzed its expression and functional relevance in CRC and observed consistent upregulation associated with poor prognosis, suggesting a potential oncogenic role in this context. Our findings also provide a basis for exploring PTPN12 as a potential biomarker across cancer types.

However, the pan-cancer analysis revealed that PTPN12 expression patterns are not uniform across malignancies—it is upregulated in several tumor types but downregulated in others, such as lung adenocarcinoma (LUAD). These divergent expression trends imply that PTPN12 may exert tissue-specific and context-dependent functions, reflecting differences in tumor-intrinsic signaling and the surrounding inflammatory microenvironment. For instance, PTPN12 has been reported to be downregulated in nasopharyngeal carcinoma and liver cancer, where it inhibits cell growth and migration (Lin et al., 2018; Liang et al., 2022), whereas in breast cancer, its loss reduces tumorigenic and metastatic potential, indicating a pro-tumorigenic role (Harris et al., 2014). In CRC, our results demonstrate that elevated PTPN12 expression is associated with unfavorable clinical outcomes, suggesting a potential role in colorectal tumor progression. Such context-dependent effects may partly relate to differences in tumor-intrinsic signaling and the surrounding tumor microenvironment across cancer types. These observations also raise the possibility that PTPN12 may represent a potential biomarker or therapeutic target in CRC, although further functional and clinical studies are required to clarify its translational relevance.

Such functional variability suggests that PTPN12’s impact may depend on the degree of inflammatory signaling, immune cell infiltration, and microenvironmental cues—factors that are particularly prominent in CRC’s inflammation-driven tumor milieu. In our genomic landscape analysis, we observed that PTPN12 exhibits multiple types of alterations across different cancer types. Although the overall frequency is low, changes in the PTPN12 locus may increase the susceptibility to tumor development. Indeed, previous studies have demonstrated that mutations in PTPN12 are associated with an increased risk of colorectal cancer susceptibility (de Voer et al., 2016). Furthermore, our comparative analysis between PTPN12-high and PTPN12-low patient groups revealed a series of recurrent mutations and copy number variations (CNVs). Notably, the PTPN12-high group exhibited a higher prevalence of 6p21.1 and 8q24.21 gains, which are known susceptibility loci associated with an increased risk of colorectal cancer (Xu et al., 2016; Tomlinson et al., 2007; Haerian et al., 2011). These findings suggest a potential link between PTPN12 dysregulation and malignant transformation, as well as an association with abnormal chromosomal structures. Beyond genomic alterations, the regulatory mechanisms underlying PTPN12 overexpression in CRC remain incompletely understood. Our analysis suggested that inflammatory signaling–related transcription factors, including the NF-κB subunit RELA, may be associated with PTPN12 expression. Given that NF-κB signaling is frequently activated during colorectal tumorigenesis, dysregulation of inflammatory pathways may contribute to the aberrant expression of PTPN12. However, further experimental studies are required to clarify the precise regulatory mechanisms.

To elucidate the mechanisms underlying PTPN12 upregulation, we predicted potential transcriptional regulators of PTPN12. Through expression correlation and survival analyses, we identified the NF-κB subunit RELA (p65) as a putative regulator of PTPN12 expression. RELA plays a pivotal role in cancer development and progression (He et al., 2019), and its functions in CRC have been extensively reported (Bakshi et al., 2022; De Simone et al., 2015; Patel et al., 2018). Our findings further support the important role of RELA in colorectal cancer and provide a basis for elucidating the molecular mechanisms underlying RELA-mediated regulation of PTPN12.

Collectively, these results suggest that aberrant activation of inflammatory signaling pathways may contribute to the upregulation of PTPN12 in CRC and subsequently influence tumor cell behavior, including proliferation, invasion, and interactions with the tumor microenvironment.

Our study also revealed the regulatory effects of PTPN12 on the TME in colorectal cancer. Through correlation and cell composition analyses, we found that PTPN12 impacts the infiltration of stromal cells, such as fibroblasts. Notably, PTPN12 is known to regulate cytoskeletal organization and cell adhesion in fibroblasts (Garton and Tonks, 1999), corroborating our findings. Furthermore, we validated our bulk RNA-seq data through single-cell analysis, which suggested that PTPN12 may contribute to the formation of an immunosuppressive microenvironment in CRC. Importantly, our *in vitro* experiments further demonstrated that PTPN12 knockdown significantly decreased PD-L1 expression at both the mRNA and protein levels, providing functional evidence that PTPN12 may participate in immune checkpoint regulation in CRC cells. These observations highlight the need to further explore the relationship between PTPN12 and the colorectal cancer tumor microenvironment.

Immunotherapy has emerged as an important treatment modality for advanced CRC. However, due to the complex crosstalk between tumor cells, stromal cells, and immune cells within the tumor microenvironment, some patients fail to benefit from immunotherapy or develop drug resistance (Chen et al., 2021; Zhao et al., 2022). Our findings provide evidence for the potential utility of PTPN12 as a novel target and biomarker for immunotherapy in CRC.

It is important to acknowledge certain limitations of our study. First, our findings are primarily based on bioinformatics analyses of publicly available datasets and small-sample validation experiments, necessitating further validation in larger real-world cohorts. Second, while we demonstrated that PTPN12 regulates key pathways such as epithelial–mesenchymal transition (EMT) and immune-related signaling *in vitro*, the lack of *in vivo* evidence limits our ability to fully delineate its functional role within the complex tumor microenvironment. The tumor–immune and tumor–stromal interactions, as well as the influence of systemic inflammatory cues, can only be reliably captured through animal models or patient-derived xenografts. Therefore, future studies will incorporate orthotopic CRC models and PTPN12 knockout mice to validate its mechanistic roles and therapeutic potential *in vivo*. Finally, comprehensive mechanistic studies and longitudinal validation in multi-center cohorts are needed to confirm the robustness and clinical relevance of our findings.

In summary, this study provides a comprehensive characterization of PTPN12 in colorectal cancer through integrated multi-omics analyses. Our findings demonstrate that PTPN12 is aberrantly expressed in CRC and is associated with unfavorable clinical outcomes. Further analyses suggest that PTPN12 may participate in the regulation of tumor-related signaling pathways and the tumor microenvironment, potentially influencing immune-related features. In addition, our results linking PTPN12 to oxaliplatin resistance indicate that it may also contribute to chemotherapy response in CRC. Collectively, these observations highlight the potential value of PTPN12 as a biomarker for CRC progression and therapeutic response. Nevertheless, further experimental and clinical studies are required to elucidate the molecular mechanisms underlying PTPN12 function and to evaluate its translational potential in CRC, and future investigations incorporating gain-of-function approaches, such as PTPN12 overexpression in CRC models, may provide additional insights into its functional role and regulatory mechanisms.

## Data Availability

The original contributions presented in the study are included in the article/Supplementary Material, further inquiries can be directed to the corresponding authors.
