# Supplementary material for: PTPN12 is a novel biomarker associated with genomic instability, therapeutic potentials, and immunomodulator in colorectal cancer
Source: Front Pharmacol. 2026 Apr 20;17:1766515. doi: 10.3389/fphar.2026.1766515 (PMC13136178; doi:10.3389/fphar.2026.1766515)
Supplement: Supplementary file 1 [file Image1.pdf]

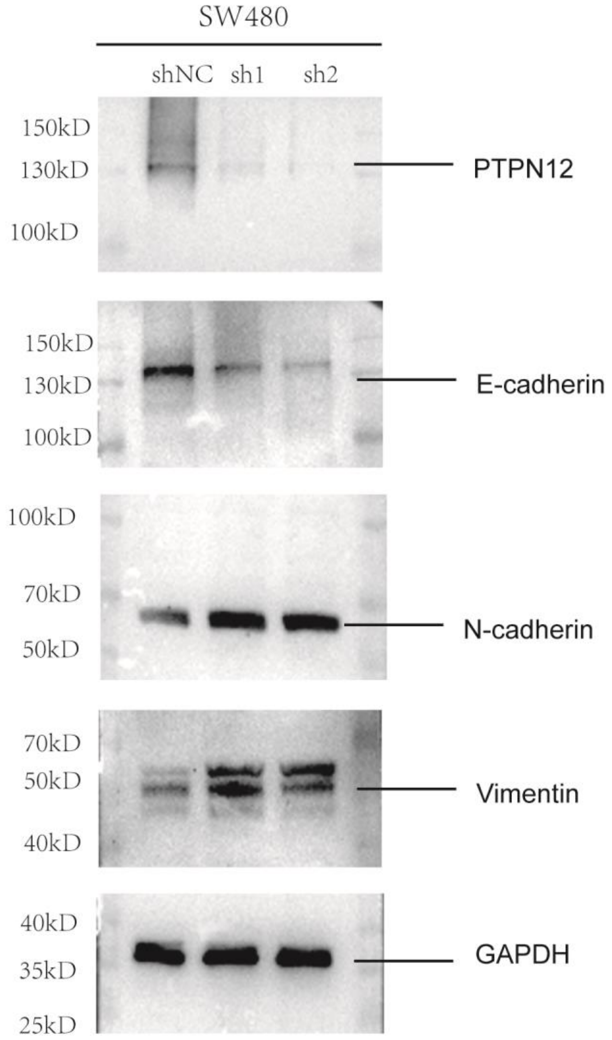

Supplementary Figure 1. Full-length uncropped Western blot images corresponding to Figure 5E. Uncropped Western blot images showing PTPN12, E-cadherin, N-cadherin, Vimentin, and GAPDH expression in SW480 cells transfected with shNC, sh1, and sh2, corresponding to the data presented in Figure 5E. Molecular weight markers (kDa) are indicated on the left.

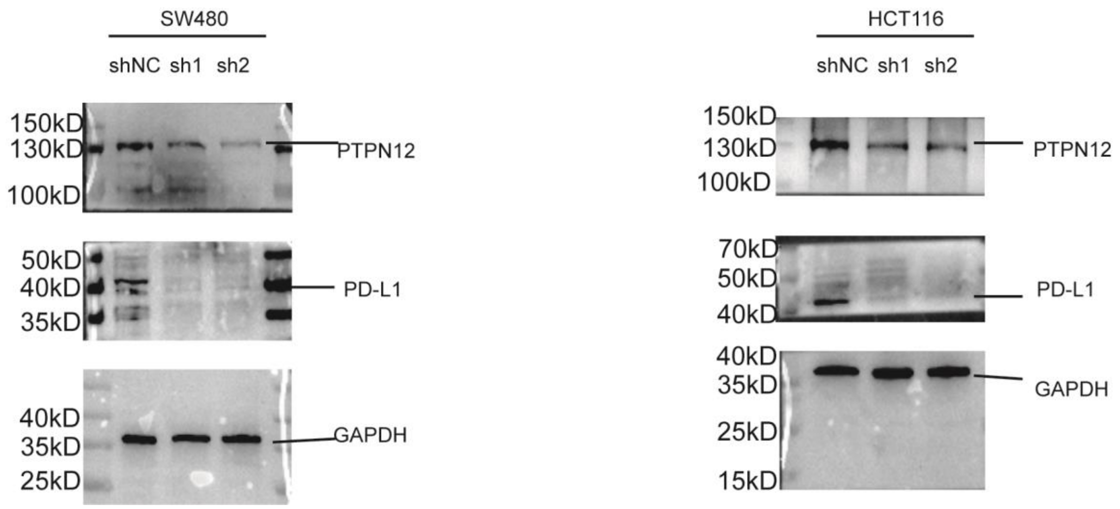

Supplementary Figure 2. Full-length uncropped Western blot images corresponding to Figure 6G. Uncropped Western blot images showing PTPN12 and PD-L1 expression in SW480 and HCT116 cells following transfection with control (shNC) or PTPN12-targeting shRNAs (sh1 and sh2). These images correspond to the cropped blots presented in Figure 6G. GAPDH served as a loading control. Molecular weight markers (kDa) are indicated on the left.

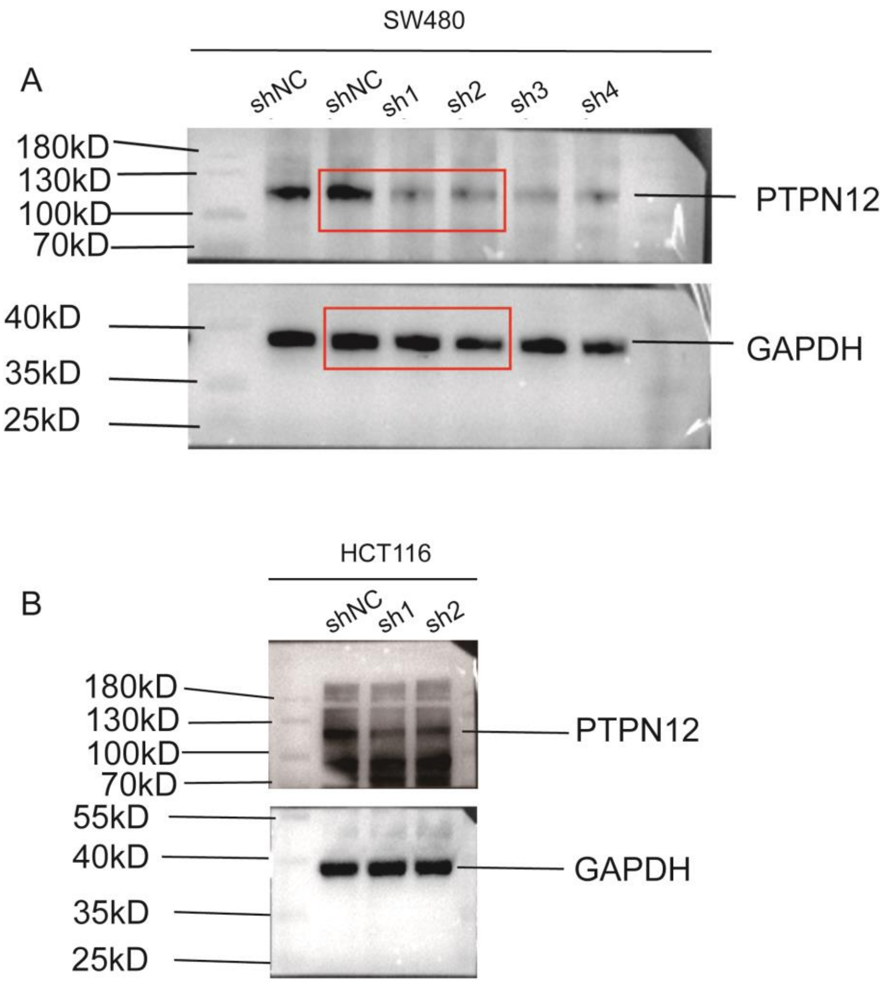

Supplementary Figure 3. Full-length uncropped Western blot images corresponding to Figures 9A and 9B. (A). Uncropped Western blot images showing PTPN12 and GAPDH expression in SW480 cells following transfection with control (shNC) or PTPN12-targeting shRNAs (sh1–sh4), corresponding to the data presented in Figure 9A; (B) Uncropped Western blot images showing PTPN12 and GAPDH expression in HCT116 cells following transfection with control (shNC) or PTPN12-targeting shRNAs (sh1 and sh2), corresponding to the data presented in Figure 9B. Molecular weight markers (kDa) are indicated on the left.

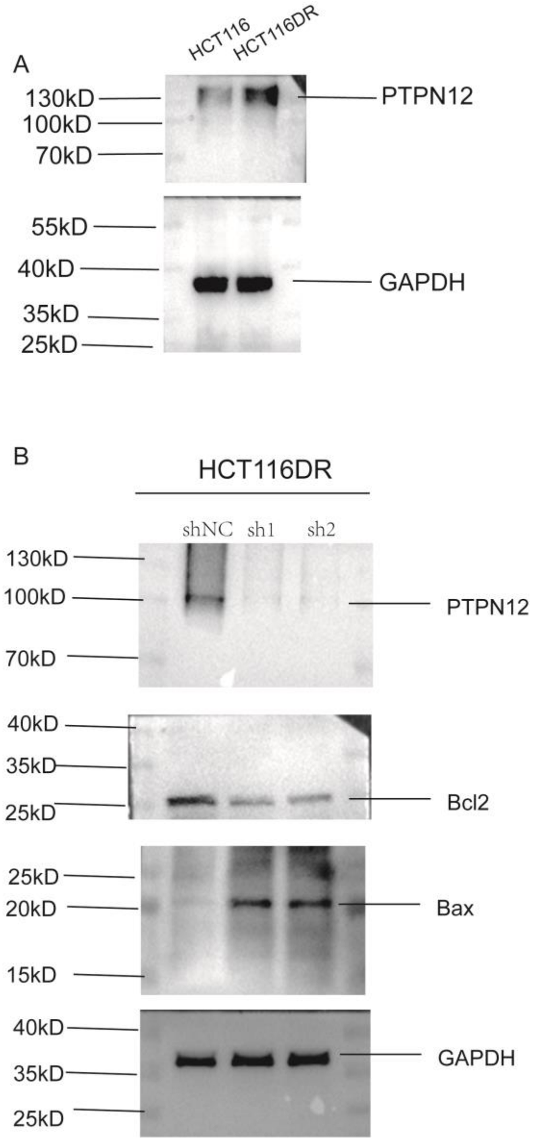

Supplementary Figure 4. Full-length uncropped Western blot images corresponding to Figures 10B and 10E. (A): Uncropped Western blot images of PTPN12 and GAPDH in parental HCT116 and drug-resistant HCT116DR cells, corresponding to the data presented in Figure 10B; (B): Uncropped Western blot images of PTPN12, Bcl2, Bax and GAPDH in HCT116DR cells following transduction with control (shNC) or PTPN12-targeting shRNAs (sh1 and sh2), corresponding to the data presented in Figure 10E. Molecular weight markers (kDa) are indicated on the left.
